# Comparison of pain neurophysiology knowledge among health sciences students: a cross-sectional study

**DOI:** 10.1186/s13104-015-1585-y

**Published:** 2015-10-22

**Authors:** Cristina Adillón, Èrik Lozano, Isabel Salvat

**Affiliations:** Unit of Preventive Medicine and Public Health, Department of Ciències Mèdiques Bàsiques, Faculty of Medicina i Ciències de la Salut, Rovira i Virgili University, Reus, Spain; Unit of Physiotherapy, Department of Medicina i Cirurgia, Faculty of Medicina i Ciències de la Salut, Rovira i Virgili University, Reus, Spain

**Keywords:** Chronic pain, Education, Neurophysiology, Pain education, Pain knowledge, Health sciences

## Abstract

**Background:**

A key tool for use in approaching chronic pain treatment is educating patients to reconceptualize pain. Thus, health professionals are fundamental to the transmission of pain information to patients. Because their understanding of pain is acquired during the educational process, the aim of this study was to compare the knowledge about pain neurophysiology in first and final-year students from three different health science programs at a single University to determine their gain in knowledge using a well-known questionnaire designed to evaluate the understanding of pain.

**Methods:**

The Neurophysiology of Pain Questionnaire (19 closed-ended questions) was administered to students in their first and final years of study in Medicine, Physiotherapy, or Nutrition. The percentage of correct responses was determined and comparisons of the results were analyzed between the programs as well as between the first and final years of study within each program. For all tests, p-values were two-sided, and results with p-values below 0.05 were considered statistically significant.

**Results:**

The participation rate was greater than 51 % (n = 285). The mean percentage of correct responses, reported as mean (SD), among the first year students was 42.14 (12.23), without significant statistical differences detected between the programs. The mean percentages of correct responses for students in their final year were as follows: Medicine, 54.38 (13.87); Physiotherapy, 68.92 (16.22); Nutrition, 42.34 (10.11). We found statistically significant differences among all three programs and between the first and final years in Medicine and Physiotherapy. A question-by-question analysis showed that the percentage of correct responses for questions related to the biopsychosocial aspects of pain was higher for students in Physiotherapy than those in Medicine.

**Conclusions:**

Students in their final years of Medicine and Physiotherapy programs know more about the neurophysiology of pain than students in their first years of these programs, however there are some questions where first years students have better results. Physiotherapy students have greater knowledge of neurophysiology of pain than Medicine students, especially the biopsychosocial aspects. Even so, their understanding may not be sufficient and does not guarantee an approach to chronic pain that will help patients reconceptualize their pain.

**Electronic supplementary material:**

The online version of this article (doi:10.1186/s13104-015-1585-y) contains supplementary material, which is available to authorized users.

## Background

The health professionals’ concept of pain is established during their formal education. However, this concept has been considerably revised in recent years. At present, it is known that pain is determined by not only the nociception caused by an injury but also other influences which, in brief, aim to protect body tissues [1]. Pain, then, is a complex process in which numerous cortical and subcortical areas participate and varies in response to the effect of numerous cognitive, motivational, or affective factors [1].

A person with chronic pain is immersed in a state of hypervigilance known as central sensitization, a state of hyperexcitability in which the threshold of pain is lowered. This hypervigilance interferes in the rehabilitation process for patients [2]. Uninformed or incorrectly informed patients believe that their pain is threatening, present a low level of tolerance to pain, and have catastrophic thoughts and fewer adaptive coping strategies [3]. Therefore, reconceptualizing pain through education is regarded as a key aid in patient treatment [4–6]. In addition, the most effective treatments for chronic pain involve a multidisciplinary approach [7], with many professionals playing important roles in educating patients.

Thus, the concept of pain held by health professionals is crucial for effective patient treatment because health professionals transmit information to patients as well as to the rest of the world.

The high incidence of chronic pain and its economic costs prompted studies analyzing the understanding of pain by students in the health sciences. These studies found that health care professionals required additional understanding in how to assist patients with their pain, and that a need existed for improving the curriculum quality for students in the health sciences [8–19]. The authors suggested the implementation of this improvement using specific, integrated curricula throughout the students’ formal education and more active methodologies [10–19]. Questionnaires were used to gather the data in some of these studies [16–19].

Although it has not been used with students, various studies have used The Neurophysiology of Pain Questionnaire [20–22] to assess pain-related knowledge. Nijs et al. [5] proposed the use of this questionnaire as a guide for clinicians during their sessions educating patients about chronic musculoskeletal pain. Traeger et al. [23] also proposed the use of this questionnaire in a study protocol as a secondary outcome assessing the efficacy of a brief educational approach for preventing chronic low back pain in at-risk individuals.

According to previously published studies, students lack important knowledge about pain at graduation [8–19], and health professionals have little understanding of the neurophysiology of pain [19], which is likely an obstacle that limits the efficiency of pain management.

Therefore, it is important to analyze the knowledge regarding the neurophysiology of pain that university students acquire. The aim of this study was to determine the level of knowledge about the neurophysiology of pain in students attending the Faculty of Medicine and Health Sciences.

## Methods

### Educational context

This cross-sectional study was conducted at the Faculty of Medicine and Health Sciences, Rovira i Virgili University in Spain. This institution offers three bachelor’s degrees relevant to this report: Degree of Medicine, Degree of Physiotherapy, and Degree of Human Nutrition and Dietetics. Approximately 280 new students enroll every academic year (125 in Medicine, 80 in Physiotherapy and 75 in Human Nutrition and Dietetics).

The three curricula are organized as traditional, independent subjects (see Table 1). The Medicine curriculum is organized around organ systems and focuses on basic science, clinical science, etc. In the Medicine program, of the six courses, the final three are most clinically relevant.

**Table 1:** Subjects related to pain taught in the Faculty of Medicine and Health Sciences

Degree	Courses (ECTS)	Type of subject (percentage of ECTS)	Subjects related to pain (ECTS)	Year	HRP
Medicine	6 (360)	Basics (26.70)	Physiology (20)	1st	1
		Instrumentals (5.50)			
		Specific (49.50)	Rehabilitation, anesthesia and pain control (3)	4th	1
			Oncologics (3)	4th	1
			Pharmacology (8)	3rt	2
		Integration (1.80)			Variable
		Clinical practice (16.50)	Clinical practice (114)		
Physio therapy	4 (240)	Basics (34.20)	Physiology (12)	1st	1
		Instrumentals (7.50)			
		Specific (29.20)	Pharmacology (5)	3rt	1.5
			Medical Pathology (7)	3rt	1
			Specific methods of physiotherapy I, II (12)	2nd	3
			General procedures in physiotherapy I (5)	2nd	2.5
		Integration (6.60)			Variable
		Clinical practice (22.50)			
Human nutrition and dietetics	4 (240)	Basics (30.60)			
		Instrumentals (18.00)			
		Specific (37.80)			
		Integration (4.10)			
		Clinical practice (9.50)			

*ECTS* European Credit Transfer and Accumulation System, *HRP* hours related to pain, *Basics* Anatomy, Physiology, Psychology, Biochemistry, *Instrumentals* Communications and Ethics, Documentation and Education, Biostatistics, *Integration* Final year project, Integrated Physiotherapy I, II, III, Integrated Nutrition I, II

### Variables

The main variable was the results from the Neurophysiology of Pain Questionnaire (see Table 2). The secondary variables were degree, current year, age, and sex.

**Table 2:** Neurophysiology of Pain Questionnaire by Moseley [20]

Question		T	F	U
1	Receptors on nerves work by opening ion channels (gates) in the wall of the nerve	#		
2	When part of your body is injured, special pain receptors convey the pain message to your brain		#	
3	Pain only occurs when you are injured		#	
4	The timing and intensity of pain matches the timing and number of signals in nociceptors (danger receptors)		#	
5	Nerves have to connect a body part to your brain in order for that body part to be in pain		#	
6	In chronic pain, the central nervous system becomes more sensitive to nociception (danger messages)	#		
7	The body tells the brain when it is in pain		#	
8	The brain sends messages down your spinal cord that can increase the nociception (danger message) going up your spinal cord	#		
9	The brain decides when you will experience pain	#		
10	Nerves adapt by increasing their resting level of excitement	#		
11	Chronic pain means that an injury hasn’t healed properly		#	
12	Nerves can adapt by making more ion channels (gates)	#		
13	Worse injuries always result in worse pain		#	
14	Nerves adapt by making ion channels (gates) stay open longer	#		
15	Second-order nociceptor (messenger nerve) post-synaptic membrane potential is dependent on descending modulation	#		
16	When you are injured, the environment that you are in will not have an effect on the amount of pain you experience		#	
17	It is possible to have pain and not know about it		#	
18	When you are injured, chemicals in your tissue can make nerves more sensitive	#		
19	In chronic pain, chemicals associated with stress can directly activate nociception pathways (danger messenger nerves)	#		

Terms in parentheses are used for patients and were not used for students in the present study

#, correct answer; T, true; F, false; U, undecided

Because the Neurophysiology of Pain Questionnaire has only been translated from English into Dutch and not Spanish [24], the Language Service of the Rovira i Virgili University translated the questionnaire into Spanish prior to the study. The original English version [20] was translated by a native-Spanish speaker into Spanish, and a native-English speaker translated the product into English. The two English versions were then compared, and the Spanish version was revised to develop the most accurate version. This version is presented in Additional file 1: Appendix 1.

This questionnaire was chosen for use because it was devised to evaluate how an individual conceptualizes pain [20]. It consists of 19 closed-ended questions with the response options of true, false, or undecided. Each correct response scored one point, whereas incorrect or undecided responses scored zero points. The questions referred to the biological mechanisms that support pain or how and why pain is perceived [25]. The Neurophysiology of Pain Questionnaire showed acceptable internal consistency (Person Separation Index, PSI = 0.84) for assessment of individuals and good test–retest reliability. Reliability of the total score for this sample was 0.971 (95 % CI 0.925–0.987) [25]. Because the questionnaire was designed for use with patients or professionals, the colloquial terms used with patients are shown in parentheses (see Table 2). However, the questionnaire in the present study used only the terms meant for professionals.

### Participants and procedure

The study population comprised undergraduate students from the Rovira i Virgili University in Spain. Participants were recruited from the Faculty of Medicine and Health Sciences during the 2012–2013 academic year as follows: first- and fifth-year students in Medicine; first- and third-year students of Physiotherapy as well as of Human Nutrition and Dietetics. Students in their fifth instead of their sixth year in Medicine were selected because they had completed the subjects related to pain knowledge, acquiring all competencies related to pain (see Table 1), and were easier to contact. During the selected academic year, the third year was the final year for students in Physiotherapy and Human Nutrition and Dietetics.

A pilot study conducted through e-mail in October 2012 had a participation rate of only 6.17 %. Therefore, the questionnaire was administered in person in the present study. All participants present in a class filled out the questionnaire simultaneously and remained anonymous. The study was conducted during March and April in 2013.

*The inclusion criterion* was to be enrolled in the Rovira i Virgili University during the 2012–2013 academic year in Medicine (first- or fifth-year students), Physiotherapy (first or third year), or Human Nutrition and Dietetics (first or third year).

### Analysis

The values of the included variables were entered into a Microsoft Office Excel 2007 spreadsheet and analyzed with Statistical Package for the Social Sciences for Windows (SPSS, version 19.0) software.

The qualitative variables were described as absolute frequencies and percentages, whereas means and standard deviations were used to describe continuous quantitative variables. Results are presented as mean (SD).

To compare the number of correct responses in the questionnaire, the percentage of correct responses (% score) was calculated with the equation ([No. of correct responses/19] × 100). The resulting means were compared using an analysis of the variance (ANOVA). If the results of the ANOVA indicated that the group differences were significant, Bonferroni corrections for multiple comparisons were used to control the type I error rate. If any condition required for the use of an ANOVA was not fulfilled (i.e., normality according to the Kolmogorov–Smirnov test and homogeneity of the variances, verified using Levene’s test), the Kruskal–Wallis test was used. When the Kruskal–Wallis test was significant, the Mann–Whitney U test was applied with Bonferroni corrections to the *p* value to compare the results among the three health sciences groups. To compare percentages of correct responses to each question, the Chi square test was used.

For all tests, p-values were two-sided, and if the value was below 0.05, the results were considered statistically significant.

### Ethical considerations

Written informed consent was obtained from all students, who were assured that the data would be processed confidentially. The study protocol was approved by the Clinical Research Ethics Committee at the University Hospital Sant Joan de Reus.

## Results

### Differences between the first and final years

Out of a total study population of 558 students, more than 51 % participated (n = 285). Of these, 40 % (n = 114) were students enrolled in the degree program for Medicine, 38 % (n = 107) in Physiotherapy, and 22 % (n = 64) in Human Nutrition and Dietetics (Nutrition). The mean age (standard deviation) was 20.9 (3.5) years and the percentage of women was 70 %.

The results from the first-year students enrolled in Medicine (n = 60), Physiotherapy (n = 65) and Nutrition (n = 47) were compared to determine if there were differences in the students’ base level of knowledge across the programs (see Table 3). The mean percentage of correct responses on the Neurophysiology of Pain Questionnaire for the first-year students was 42.14 (12.23), with no statistically significant differences detected among the three degree programs (p = 0.847).

**Table 3:** Students’ demographical data and results on the Neurophysiology of Pain Questionnaire

	Medicine	Physiotherapy	Nutrition
First-year students			
n	60	65	47
Age (years)	18.9 (1.5)	19.8 (3.1)	20.4 (3.5)
Gender (women %)	75.0 %	53.8 %	76.6 %
NPQ score (%)	42.2 (13.2)	42.7 (11.7)	41.3 (10.1)
Final-year students			
n	53	42	17
Age (years)	22.9 (1.8)	22.4 (3.3)	23.4 (6.9)
Gender (women %)	73.6 %	69.0 %	82.4 %
NPQ score (%)	54.4 (13.9)*	68.9 (16.2)*	42.3 (10.1)

Values indicate percentage of correct responses mean (standard deviation)

*NPQ* Neurophysiology of Pain Questionnaire

* p < 0.05 (Mann–Whitney U test, analysis between the first and final years)

When the results of the final-year students were analyzed (see also Table 3), we found that the mean of the correct responses to the Neurophysiology of Pain Questionnaire was 58.13 (16.90) and that the differences in the results between degrees reached statistical significance (Medicine vs. Physiotherapy p < 0.001, Medicine vs. Nutrition p < 0.05, and Physiotherapy vs. Nutrition p < 0.001).

The analysis of the differences in the percentage of the correct responses between the first and final years revealed statistically significant differences for students enrolled in Medicine and Physiotherapy (p < 0.001) but not in Nutrition (p = 0.346).

On the other hand, the results were compared by gender.

### Differences between the two sexes

As the results of first year students show no statistically significant differences, we decided to group the three undergraduate degrees (Medicine, Physical therapy and Nutrition) to do the analysis. From a total study population of 172 students in first year degree courses, 116 (67 %) were women and 56 (33 %) were men. The mean percentage of correct responses was 41.66 (13.20) for men and 37.00 (11.29) for women with statistically significant differences between sexes (ANOVA, p < 0.05).

When the results of the final-year students were analysed (see Table 4), we found that Physical Therapy was the only degree to show statistically significant differences between sexes (Mann–Whitney U test, p < 0.01).

**Table 4:** Neurophysiology of Pain Questionnaire between genders in the final year

	Men	Women	P
Medicine			
n (%)	15 (28)	39 (72)	0.540
NPQ score	55.78 (12.71)	53.85 (14.41)	
Physiotherapy			
n (%)	13 (31)	29 (69)	0.006*
NPQ score	78.95 (13.59)	64.43 (15.45)	
Nutrition			
n (%)	3 (18)	14 (82)	0.197
NPQ score	50.88 (3.04)	41.73 (10.42)	

Values indicate percentage of correct responses mean (standard deviation)

*NPQ* neurophysiology of pain questionnaire

* p < 0.05 (Mann–Whitney U test)

### Question-by-question analysis

A more detailed analysis was then undertaken to determine which questions obtained the highest or lowest percentage of correct responses from the students in the final years of Medicine and Physiotherapy (Nutrition was excluded from the analysis because no differences were found in the questionnaire results between the first and final years in this program).

The question that obtained the highest percentage of correct responses in the final years was number 3, “Pain only occurs when you are injured” (>95 %). The question that obtained the lowest percentage of correct responses was number 2, “When part of your body is injured, special pain receptors convey the pain message to your brain” (<10 %).

The percentage of correct responses in the final years of the Physiotherapy and Medicine programs were compared and statistically significant differences were found (p < 0.05) for question 5 (“Nerves have to connect a body part to your brain in order for that body part to be in pain”), question 6 (“In chronic pain, the central nervous system becomes more sensitive to nociception”), question 7 (“The body tells the brain when it is in pain”), question 9 (“The brain decides when you will experience pain”), question 17 (“It is possible to have pain and not know about it”), and question 18 (“When you are injured, chemicals in your tissue can make nerves more sensitive”) of the Neurophysiology of Pain Questionnaire (see Table 5, in which the 19 questions are grouped based on their content: biological mechanisms that support pain and how and why pain is perceived).

**Table 5:** Percentage of correct responses on the Neurophysiology of Pain Questionnaire in the final year

	Medicine	Physiotherapy
Biological mechanisms		
Q1	57.41	59.52
Q2	3.70	9.52
Q8	64.81	76.19
Q10	33.33	42.86
Q12	75.93	76.19
Q14	83.33	73.81
Q15	38.89	54.76
Q18*	83.33	97.62
How and why pain is perceived		
Q3	96.30	100.00
Q4	46.30	64.29
Q5*	14.81	52.38
Q6*	40.74	83.33
Q7*	5.56	52.38
Q9*	11.11	57.14
Q11	68.52	83.33
Q13	96.30	97.62
Q16	88.89	78.57
Q17*	35.19	59.52
Q19	88.89	90.48

*Q* question

* p < 0.05 (Chi square test)

A comparison among students in the same program from the first and final years showed that in most cases the percentage of correct responses to each question increased. However, in some cases, a decrease was observed (see Table 6). For question 7, “The body tells the brain when it is in pain,” the percentage of correct responses was significantly lower for the final-year than for the first-year students in Medicine (p < 0.05).

**Table 6:** Neurophysiology of Pain Questionnaire questions responded to correctly by more first than final-year students

Question		Medicine	Physiotherapy
1	Receptors on nerves work by opening ion channels in the wall of the nerve		x
2	When part of your body is injured, special pain receptors convey the pain message to your brain	x	x
5	Nerves have to connect a body part to your brain in order for that body part to be in pain	x	
7	The body tells the brain when it is in pain	x*	
9	The brain decides when you will experience pain	x	
11	Chronic pain means that an injury hasn’t healed properly	x	

*x,* the number of correct responses by final-year students is lower than first-year students in the same program

* p < 0.05 (Chi square test)

## Discussion

This study used the Neurophysiology of Pain Questionnaire to compare the level of knowledge about the neurophysiology of pain among students enrolled in the first and final years of three health sciences degree programs at the Faculty of Medicine and Health Sciences. We found that knowledge of the neurophysiology of pain for final-year students was higher than first-year students enrolled in the Medicine and Physiotherapy programs, but not in the Nutrition program. However, this latter program did not specifically deal with pain, whereas the other two programs did. We also found that final (third)-year students in Physiotherapy had higher scores on the Neurophysiology of Pain Questionnaire than final (fifth)-year students in Medicine. A question-by-question analysis revealed that a higher percentage of Physiotherapy students gave correct responses to question numbers 5, 6, 7, 9, 17, and 18 of which questions 5, 6, 7, 9 and 17 examined knowledge of “How and why pain is perceived.” Therefore, Physiotherapy students not only displayed the overall greatest knowledge of the neurophysiology of pain, but they specifically understood the biopsychosocial aspects of pain the best of the three groups.

Another result obtained in this study supports this interpretation. We observed that the number of students who responded correctly to some questions decreased between the first and the final years in the programs. This was particularly the case for students in the Medicine program and for the questions in the section “How and why pain is perceived.” For example, on question 7, which focuses on an essential, basic aspect of pain, the difference between the percentage of students who responded correctly in the first and final year was statistically significant. Responding incorrectly or not responding to this question meant that the students confused the concepts of pain and nociception.

It would be interesting to use the data obtained in this study to determine whether final-year students’ knowledge of pain is sufficient to guarantee that chronic pain will be treated appropriately. This cannot be determined on the basis of other publications. Unfortunately, no gold standard exists for such comparisons, and it would be difficult to compare our results with those of Moseley [20] or Catley et al. [25] because neither study reports the method used to count the correct responses on the Neurophysiology of Pain Questionnaire. If we assume that Moseley counted the responses using the same method used in the present study, then the results of our final-year Medicine students would fall between those reported in that study for trained doctors (67 %) and for doctors who received no specific training in pain (33 %). However, the results of final-year students in the Physiotherapy program would be markedly higher than those reported by Moseley for trained (29 %) and untrained (11 %) physiotherapists.

It is even more difficult to compare our results with those of Catley et al. [25], who slightly revised the questionnaire to adapt it to patients. The data reported in that study reflect the effects of educational intervention, with correct response values of 26.40 % (13.50) before and 63.20 % (15.70) after an educational program.

Despite the inability to directly compare our results with those of other published studies, the students’ scores are currently too low. We believe that the knowledge of the neurophysiology of pain required to assist patients to reconceptualize pain using cognitive interventions should be higher than that found in the present study.

Our results are consistent with those of studies analyzing the knowledge of pain for students in different curricula and finding that knowledge insufficient [8–19]. The percentage of time spent on specific training in the knowledge of pain and pain management approaches is insufficient both in Medicine and Physiotherapy (see Table 1) and far below the credits those curricula propose. Moreover, while other courses have specific topics, the instruction method in pain training is through the inclusion of pain issues within other subjects; for instance, in respiratory diseases in both medicine and physiotherapy there is a subject linked with clinical practice; 6 ECTS, 3 ECTS, respectively. This way of teaching tends to produce a fragmented, ineffectual understanding of pain [13].

As far as teaching methodology is concerned, several previous studies have detected a lack of knowledge of pain and have proposed various teaching strategies to combat this deficit. Tauben et al. and Vadivelu et al. suggested increasing the number of hours in the curriculum spent teaching students how to handle pain [8, 14]. Murinson et al. and Argyra et al. focused on approaching the emotional aspects of handling pain [9, 19], and Chen et al. and Bair introduced the importance of communicating with and educating patients, key aspects in approaching pain management [10, 15]. Merlin et al. [12] and Mezei et al. [13] recommend integrating pain topics into health science curricula. Although the Physiotherapy program examined in the present report devoted no more hours than the Medicine program to the study of pain, the Physiotherapy program used active tools to encourage students to reflect on the concept of pain. (We reported elsewhere how students produced an educational instrument capable of helping patients understand pain as part of a subject focusing on the biopsychosocial aspects of pain [26]).

To improve pain training we suggest: firstly, increasing the time spent in pain education to 25 h, with clinical elective pain courses from 177 to 318 h; secondly, that the pain training is the same across other subjects, with specific subjects and integrated into case-based clinical experiences and during clinical clerkships [13]. However, that would not be sufficient. It is necessary to develop care plans based on the biopsychosocial model. Also, students will need not only clinical knowledge but also to be prepared to address the professional, personal, and ethical challenges that arise in caring for those in pain (promotion of compassionate practices, fostering reflective and interactive case-based learning, reflective work…).

We could use existing FMCS resources to improve pain training in instrumental subjects for instance, dealing with ethical issues related to patients with chronic pain. Also, the portfolio could be used to allow students to explore and record their experiences of the impact of pain and to begin to examine their own responses to this problem. In addition, an inter-professional course on pain for physical therapists and medical students could be organized. A committee could decide which version of published pain education program would be determined most relevant.

Some of the study results are amazing. We found that men had a higher percentage of correct answers than women (in first year degrees and in Physical Therapy and in final year). These results suggest that men perceive better the biopsychosocial aspects of pain. The lowest percentage for correct responses was provided for Question number 2. That is also an amazing result if we consider what Catley et al. [25] state about this question: shows excessive negative outfit, suggesting they were overly predictable with Q5, Q6, Q10, Q13, Q17. On the other hand, this is one of the questions that was left in the revised questionnaire [25]. We believe this question might be misunderstood because of the words “special pain receptors”. The subjects understand “nociceptors”, but the question says “pain receptors”, which is misleading because pain is a brain construct.

This study had several limitations. Although our conclusions cannot be generalized to other programs, it would be interesting to know the extent to which students in other health science degree programs, such as in nursing or psychology, understand pain. Another limitation of this study was the questionnaire that we used. Despite the widespread use of the Neurophysiology of Pain Questionnaire, its psychometric properties have only been partially investigated. Although this questionnaire offers valid and reliable measures for patients with chronic pain [24] and chronic spinal pain [25], its psychometric properties have not been explored in students. Moreover, because the results for students in Physiotherapy were better than those in Medicine, the Neurophysiology of Pain Questionnaire may be biased toward physiotherapy, even though Moseley specifically states that the design was based on postgraduate exams testing the knowledge of pain in a medicine degree program.

## Conclusions

In general, students in the final years of their degree programs in Medicine and Physiotherapy at the Faculty of Medicine and Health Science in Rovira i Virgili University had greater knowledge of the neurophysiology of pain than students in their first year, with the exception of certain basic aspects of pain neurophysiology in which we found a setback. Third-year students in the Physiotherapy program had higher scores on the Neurophysiology of Pain Questionnaire than fifth-year students in the Medicine program. We also found greater knowledge of the biopsychosocial aspects of pain for students in their final year of the Physiotherapy degree compared with those in Medicine or Nutrition. Despite this, the understanding of the students in Physiotherapy may not be sufficient and does not guarantee that their approach to chronic pain will be reconceptualized through additional educational intervention.

This study is consistent with those studies aiming to improve the training of students in health sciences programs in the treatment of pain to meet the social needs in this field. Specifically, the present study identifies the lack of training for students on the psychosocial aspects of pain, which are essential for treating patients with chronic pain and for assisting those patients to reconceptualize pain.

## Additional file

10.1186/s13104-015-1585-y Cuestionario sobre la neurofisiología del dolor.
