# Supplementary material for: Comparison of pain neurophysiology knowledge among health sciences students: a cross-sectional study
Source: BMC Res Notes. 2015 Oct 22;8:592. doi: 10.1186/s13104-015-1585-y (PMC4619188; doi:10.1186/s13104-015-1585-y)
Supplement: Supplementary file 1 — 10.1186/s13104-015-1585-y Cuestionario sobre la neurofisiología del dolor. [file 13104_2015_1585_MOESM1_ESM.docx]

| **Appendix 1 Cuestionario sobre la neurofisiología del dolor.** | | | |
| --- | --- | --- | --- |
| Preguntas | **V** | **F** | **NS** |
| Los receptores de los nervios actúan abriendo los canales iónicos (puertas) de las paredes del nervio. |  |  |  |
| Cuando sufre una lesión en alguna parte del cuerpo, los receptores específicos del dolor transmiten el mensaje de dolor al cerebro. |  |  |  |
| El dolor únicamente se produce cuando sufre una lesión. |  |  |  |
| La duración y la intensidad del dolor corresponden a la duración y cantidad de las señales de los nociceptores (receptores del peligro). |  |  |  |
| Los nervios deben conectar una parte del cuerpo al cerebro para que esa parte del cuerpo sienta dolor. |  |  |  |
| Cuando hay dolor crónico, el sistema nervioso central es más sensible a la nocicepción (mensajes de peligro). |  |  |  |
| El cuerpo informa al cerebro cuando siente dolor. |  |  |  |
| El cerebro envía mensajes a través de la médula espinal que pueden aumentar la nocicepción (mensaje de peligro) que asciende por la médula espinal. |  |  |  |
| El cerebro decide cuándo sentirá dolor. |  |  |  |
| Los nervios se adaptan aumentando su nivel de excitación en reposo. |  |  |  |
| El dolor crónico significa que una lesión no se ha curado adecuadamente. |  |  |  |
| Los nervios pueden adaptarse creando más canales iónicos (puertas). |  |  |  |
| Las lesiones más graves siempre causan dolor más intenso. |  |  |  |
| Los nervios pueden adaptarse haciendo que los canales iónicos (puertas) permanezcan abiertos más tiempo. |  |  |  |
| El potencial de membrana postsináptico del nociceptor de segundo orden (nervio mensajero) depende de la modulación descendente. |  |  |  |
| Cuando sufre una lesión, el ambiente en el que se halla no influirá en la cantidad de dolor que siente. |  |  |  |
| Es posible tener dolor y no darse cuenta. |  |  |  |
| Cuando sufre una lesión, las sustancias químicas del tejido pueden hacer que los nervios estén más sensibles. |  |  |  |
| Cuando hay dolor crónico, las sustancias químicas relacionadas con el estrés pueden activar directamente las vías de la nocicepción (nervios transmisores del peligro). |  |  |  |
| V, verdadero; F, falso; Ns, no sabe | | | |
